# *In vitro* engineering of human 3D chondrosarcoma: a preclinical model relevant for investigations of radiation quality impact

**DOI:** 10.1186/s12885-015-1590-5

**Published:** 2015-08-08

**Authors:** Dounia Houria Hamdi, Sofia Barbieri, François Chevalier, Jean-Emmanuel Groetz, Florence Legendre, Magali Demoor, Philippe Galera, Jean-Louis Lefaix, Yannick Saintigny

**Affiliations:** 1 LARIA-IRCM-DSV-Commissariat à l’Energie Atomique et aux Energies Alternatives, CIMAP, GANIL, Bd Henri Becquerel, BP 55027, 14076 Caen, cedex 05 France; 2 Università degli Studi di Pavia, Pavia, Italy; 3 UMR6249-Université de Franche-Comté, Besançon, France; 4 EA4652-Normandie Université, Caen, France

## Abstract

**Background:**

The benefit of better ballistic and higher efficiency of carbon ions for cancer treatment (hadron-therapy) is asserted since decades, especially for unresectable or resistant tumors like sarcomas. However, hadron-therapy with carbon ions stays underused and raises some concerns about potential side effects for patients. Chondrosarcoma is a cartilaginous tumor, chemo- and radiation-resistant, that lacks reference models for basic and pre-clinical studies in radiation-biology. Most studies about cellular effects of ionizing radiation, including hadrons, were performed under growth conditions dramatically different from human homeostasis. Tridimensional *in vitro* models are a fair alternative to animal models to approach tissue and tumors microenvironment.

**Methods:**

By using a collagen matrix, standardized culture conditions, physiological oxygen tension and a well defined chondrosarcoma cell line, we developed a pertinent *in vitro* 3D model for hadron-biology studies. Low- and high-Linear Energy Transfer (LET) ionizing radiations from GANIL facilities of ~1 keV/μm and 103 ± 4 keV/μm were used respectively, at 2 Gy single dose. The impact of radiation quality on chondrosarcoma cells cultivated in 3D was analyzed on cell death, cell proliferation and DNA repair.

**Results:**

A fair distribution of chondrosarcoma cells was observed in the whole 3D scaffold. Moreover, LET distribution in depth, for ions, was calculated and found acceptable for radiation-biology studies using this kind of scaffold. No difference in cell toxicity was observed between low- and high-LET radiations but a higher rate of proliferation was displayed following high-LET irradiation. Furthermore, 3D models presented a higher and longer induction of H2AX phosphorylation after 2 Gy of high-LET compared to low-LET radiations.

**Conclusions:**

The presented results show the feasibility and usefulness of our 3D chondrosarcoma model in the study of the impact of radiation quality on cell fate. The observed changes in our tissue-like model after ionizing radiation exposure may explain some discrepancies between radiation-biology studies and clinical data.

**Electronic supplementary material:**

The online version of this article (doi:10.1186/s12885-015-1590-5) contains supplementary material, which is available to authorized users.

## Background

Emerging protocols of radiation-therapy (RT) with charged particles (protons or heavier ions than helium ions), in advanced medical facilities have widely changed the way of thinking about local tumor control and impact on healthy tissues. Indeed, charged particle-therapy (hadron-therapy) has the advantage of an excellent beam ballistic and a minimal exit dose after energy deposition in the target volume, and hence better sparing of critical structures in the vicinity of the tumor [1]. Unlike photons, protons and heavy ions exhibit a depth-dose distribution profile characterized by the Bragg peak, a sharp rise in energy deposition at the end of their range with a steep dose falloff downstream. However, the ratio of dose at the Bragg peak to that in the entrance region is higher for heavy ions [2]. Furthermore, compared to photons and protons, heavy ions have a higher Linear Energy Transfer (LET). Because high-LET radiation is densely ionizing, the correlated DNA damages within one cell occur more often so that it becomes more difficult for the cell to repair the damage, leading to a markedly increased efficiency of cell killing. In addition, heavy ions have less dependency on cell cycle and oxygen tension. Indeed, a particle beam with a high-LET (LET ~100 +/− 20 keV/μm) is required to meet an optimal biological effectiveness [1]. Thus, RT with heavy ions such as carbon ions represents an attractive radiation modality, which combines the physical advantages of protons, with a higher radiobiological effectiveness. Thanks to such improved biological effectiveness, these technologies are expected to reduce frequency and severity of radiation morbidity. However, the tremendous amount of combination of radiation quality (LET, energy, dose rate, dose) and tissue biological status (co-morbidity factors, genetic background, O_2_ tension) does not simplify the building of a relevant model for exposure of healthy tissues or tumors during RT [3]. Therefore, it is necessary to develop new tools in order to optimize the use of hadron beams in cancer therapy either in the development of new instruments for beam control and dosimetry or in the understanding of the biological effects of hadrons on healthy tissue and various kinds of tumor.

Chondrosarcoma (CHS) is a malignant skeletal tumor with cartilaginous differentiation (dissimilar from other primary skeletal tumors) and represents the second most common primary bone tumor in adults, generally arising in the fourth decade. It is a heterogeneous group of tumors that have in common the production of chondroid matrix. Conventional CHS subgroup represents ~ 85 % of total cases and can be subdivided in low-grade (I), intermediate-grade (II) or high-grade (III) based on histology [4]. Primary treatment is surgical but, due to the location of tumors close to critical structures (abdomen, cranial and spinal nerves), the complete resection is rarely possible. Indeed, CHS is considered as a chemo- and radiation-resistant cancer, needing high dose RT in inoperable or incompletely resected tumors [2]. Hadron-therapy has been applied to the treatment of low- and intermediate-grade CHS at different locations, with very promising results [2, 5, 6]. However, prospective randomized trials comparing different RT modalities are still needed to validate the superiority of one treatment for a given indication. The understanding of the impact of low- versus high-LET beams on normal and tumor tissues is, then, important to enhance the knowledge and serve clinical use of hadrons.

To date, most current CHS animal models consist in the subcutaneous xenografting of CHS cell lines or human tumor tissue [7]. Three orthotopic CHS mouse models were published recently [8–10] using CHS human cell lines, but there is no transgenic mouse model for this disease. Considering the lack of radiation-biology studies on CHS, in contrast with the existence of a subset of medical data confirming the effectiveness of hadrons, and the absence of an *in vivo* reference model, we developed a new CHS three-dimensional (3D) model in order to mimic in *vivo* microenvironment. Indeed, over the past sixty years, two-dimensional monolayer cells (2D) cultured in atmospheric oxygen tension (20 % O_2_, referred as normoxia) have been considered as a gold standard in cell biology and more specifically in radiation-biology studies. However, the 3D environment [11] and oxygen tension [12] have a major impact on cellular response to ionizing radiations (IRs). Basic 3D cultures of CHS cells have been previously used for drug testing but chondrogenic properties of type I/III collagen [13], and physioxic culture conditions were not taken into account as demonstrated previously [14, 15]. Indeed, in a previous study, P. Galera’s team proposed a new 3D cartilage model (3DCaM), relevant for arthritis analysis [15]. They used a 3D scaffold composed of cross-linked type -I and -III collagen, and successfully handled this matrix with Articular Chondrocytes (AC), isolated from human donors. As compared with conventional *in vitro* 2D culture, they showed the advantages of this 3D scaffold, in association with chondrogenic factors and physiological oxygen tension (2 % O_2_, referred as physioxia), to allow cell re-differentiation and natural cartilage matrix synthesis. It should be noted that cartilage is the only avascular tissue of human body explaining the low O_2_ tension of this tissue [16, 17].

Using the most characterized grade II CHS cell line (SW1353), a standardized chondrocyte medium, a chondrogenic factor (BMP-2), physiological oxygen tension (2 % O_2_) and the same collagen scaffold [15, 18], we report here the first 3D CHS model (3DCM) applied to radiation-biology studies. We used two different IRs; accelerated ^18^O ion beam as a high-LET radiation, comparable to the LET of carbon ions beam delivered into the tumor volume (Spread-Out Bragg Peak, SOBP) during hadron-therapy [19], and X-rays as a low-LET radiation (control). We used a single 2 Gy dose and LET distribution profile of ions was calculated in order to ascertain a homogenous irradiation of 3DCM. Radiation-induced cell death was assessed with our 3DCM and canonical clonogenic assay in 2D culture as a reference. Ki67 proliferation index and gamma-H2AX kinetic were carried out to demonstrate the feasibility and the proof of usefulness of 3DCM in hadron-biology and the impact of radiation quality on proliferation and DNA double strand breaks (DSBs) repair.

## Methods

### Reagents and antibodies

SW1353 CHS cell line, human Articular Chondrocytes (AC) and the following culture media were purchased from CellSystems (Troisdorf, Germany); Chondrocyte Growth Medium w/o Phenol Red (#411PR-500), Chondrocyte Basal Medium w/o Phenol Red w/o FBS (#410PR-500) and Chondrocyte Growth Medium w/o Phenol Red w/o FBS (#411FPR-500). Collagen scaffolds were bought from Symatese Biomateriaux (Chaponost, France), BMP-2 (Bone Morphogenic Protein-2) from R&D Systems (Minneapolis, USA), Toxilight™ assay from Lonza (Basel, Switzerland), Digitonin from Promega (Madison, USA), T-PER (Tissue Protein Extraction Reagent), Halt Protease Inhibitors Cocktail 100 X, Halt Phosphatase Inhibitors single-use Cocktail 100 X, anti-GAPDH (#11335232), anti-rabbit HRP-coupled (#31460) and anti-mouse HRP-coupled (#31430) antibodies from Thermoscientific (Waltham, USA), anti-H2AX phosphoserine 139 (#05-636) and anti-Ki67 (#AB9260) antibodies, ECL classico/crescendo and Accutase™ from Merck Millipore (Darmstadt, Germany), DAB (Diaminobenzidine) from Life technologies (Carlsbad, USA).

### Cell cultures

All the data reported in this manuscript have been collected from commercially available human healthy chondrocytes and CHS cells used in compliance with the Helsinki Declaration. CellSystems Company (Troisdorf, Germany) and its supplier, Cell Applications Company (San Diego, USA), follow bioethics guidelines to comply with the Helsinki Declaration.

The use of human cells by our Institute and Laboratory was approved by the French Ministry of Research under the CODECOH reference: DC-2008-228.

From Dulbecco's Modified Eagle's Medium (DMEM) supplemented with 2 mM L-glutamine, 10 % FBS and antibiotics (100 U/mL of penicillin V, 100 μg/mL of streptomycin), cells were gradually adapted to a standardized Chondrocyte Growth Medium (#411PR-500). Cells were checked for Mycoplasma contamination and aliquots frozen for further experiments. During cell expansion (2D culture), cells were seeded at 1.3 × 10^4^ cells/cm^2^ in 75 cm^2^ culture flasks and maintained at 37 °C in a humid atmosphere (95 % air, 5 % CO_2_). Instead of trypsin, Accutase™ was used as a cell detachment reagent. SW1353 were passaged twice a week, not more than ten times.

**Fig. 1:**
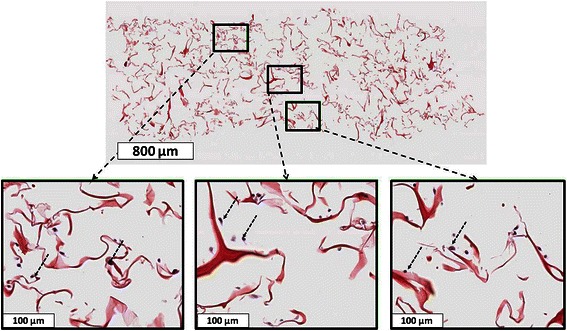
Homogenous cell distribution in the 3D scaffold. Top: A representative image of a paraffin-embedded, HES colored 8 μm section of a 3DCM. Bottom: Magnified images (*from top image*) corresponding to the proximal (*left*), internal (*middle*) and distal (*right*) zones of the scaffold. The collagen fibers are colored in pale red and the cells, indicated with dotted arrows, in violet

For 3D experiments, SW1353 cells were grown in collagen scaffolds (Fig. 1) as described previously for Articular Chondrocytes (AC) [15, 18]. These scaffolds were prepared by Symatese Biomatériaux (Chaponost, France) and are composed of native type I collagen (90–95 %) and type III collagen (5–10 %) from calf skin. They were cross-linked using glutaraldehyde to increase their stability and sterilized with γ-irradiation [18, 20]. They were punched with a skin biopsy punch (Laboratoires Stiefel, France) as discs of 5 mm diameter and 2 mm thickness (which corresponds to a volume of 0.04 cm^3^). Their pore size is around 100 nm [18].

P. Galera’s team model was adapted for radiation-biology experiments but instead of DMEM, we used a 3D Chondrocyte Medium (#411FPR-500) supplemented with 2 % FBS. Briefly, cells were seeded onto the scaffold at 4 × 10^5^ cells/scaffold in 96-well culture plates and incubated at 37 °C and 5 % CO_2_. One hour later, 100 μL of the previous medium supplemented with BMP-2 (50 ng/mL) were added to the well and cells were incubated in physioxia (2 % O_2_) in a Heracell™ 150i Tri-Gas Incubator, for 7 days to obtain a 3DCM. The medium was changed twice a week.

### Cell cycle analysis

Cell cycle distribution analysis was performed in 2D culture. SW1353 cells were plated at subconfluency in T25 culture flasks then placed in the incubator for 6 h. Cells were then harvested and centrifuged at 800 rpm for 5 min. Cell pellet was washed in PBS, fixed in ethanol 75° then stored at 4 °C until analysis. Briefly, cells were centrifuged at 2000 rpm for 5 min, and the cell pellet resuspended in PBS before staining. The remaining pellet was gently resuspended in 500 μL DNA Prep Stain and 50 μL DNA Prep LPR (DNA Prep Reagent kit, Beckman Coulter). Samples were incubated in the dark for 15 min and a minimum of 1 × 10^4^ cells per sample were analyzed using GALLIOS flow cytometer (Beckman Coulter, Passadena, USA). FlowJo analyzing software (Ashland, USA) was used. Experiments were repeated four times and data expressed as mean ± Standard Error on the Mean (S.E.M.).

### Low-LET irradiation

Low-LET radiation exposure was performed, either at the CLCC (Centre de Lutte Contre le Cancer) François Baclesse (Caen, France) or at Cyceron facility (Caen, France). We used respectively, a Saturne 15 (15 MV, 6 mA, Siemens) medical linear accelerator producing X-rays or an X-RAD 225 Cx (225 kV, 13 mA, PXi) research X-rays generator intended to cellular and small animal irradiation. The X-RAD 225 Cx is characterized by a first half value layer of 0.9 mm of copper. X-rays of this beam produce low energy secondary electrons with dE/dX ranging from 0.26 to 2.25 keV/μm with a mean value of 1.65 keV/μm (instead of 0.2 keV/μm for MV beams). Absolute dosimetry of the irradiator was performed following AAPM TG-61 protocol [21] and the dose delivered to cellular samples was measured thanks to ionization chamber measurements and thermo luminescent dosimeters (TLD). Except for survival curves, the canonical single dose fraction in conventional radiotherapy of 2 Gy was used for all experiments at a 2 Gy/min dose rate.

**Fig. 2:**
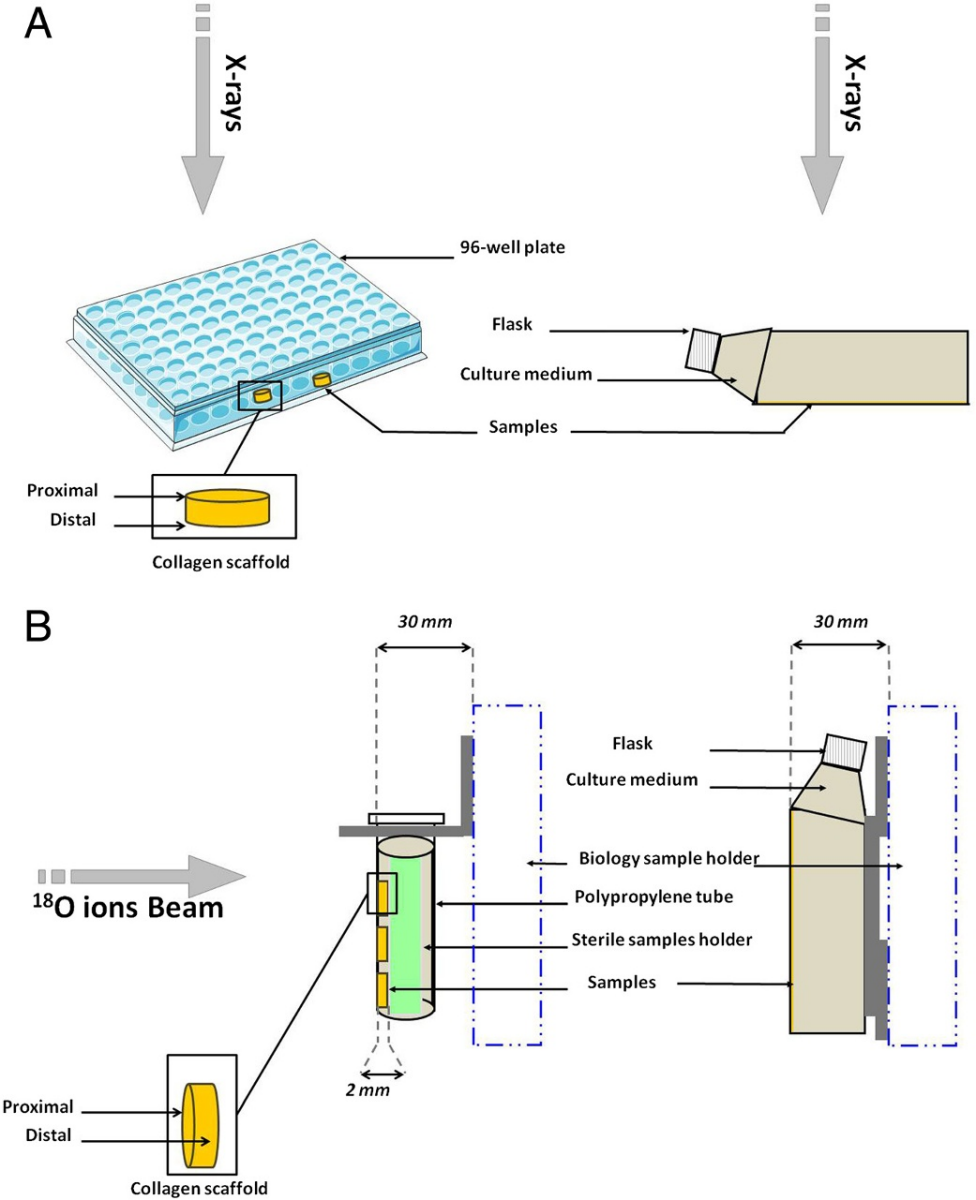
Schematic representation of the irradiation set-up for 2D cells and 3DCM. Panel **a**: X-rays irradiation set-up. Panel **b**: heavy ions irradiation set-up

Irradiation of 2D cells was performed as follows: cells were seeded 48 h prior to irradiation, at a density of 2.4 × 10^4^/cm^2^ in 12.5 cm^2^ culture flasks (BD Falcon™), so that they reach subconfluency at the time of irradiation. One day later, they were incubated in a Tri-Gas Incubator (2 % O_2_) for physioxic irradiation environment. Immediately prior to irradiation, flasks were completely filled with 2D Chondrocyte Basal Medium (#410PR-500) previously balanced with 2 % O_2_, and then sealed in order to maintain a constant oxygen tension. Then, they were placed on a horizontal plate within the X-ray generator (Fig. 2a). Mock-irradiated cells were handled in the same conditions without being irradiated. Following irradiation, cells were maintained in the Tri-Gas Incubator (2 % O_2_) in fresh Growth Medium previously balanced with 2 % O_2_ for further cell survival experiments.

Irradiation of 3DCM was performed as follows: the 96-well plates containing the 3DCM were sealed with parafilm in order to maintain 2 % O_2_ tension during irradiation and put on the horizontal plate within the X-ray generator (Fig. 2a). Mock-irradiated cells were handled in the same conditions without being irradiated. Following irradiation, medium was changed. Samples were collected at different time points, rinsed once in Dulbeccos’s PBS then stored either at −80 °C for western blot analysis or formalin-fixed for immunohistochemistry-paraffin (IHC-p). Culture supernatant (100 μL) was also collected and stored at −80 °C for cell toxicity assay.

### High-LET irradiation

High-LET radiation exposure was performed using the D1 IRABAT high-energy scanning beam line at the Grand Accélérateur National d’Ions Lourds (GANIL, Caen, France). The dosimetry and calibration beam was done by CIMAP (Centre de Recherche sur les Ions, les Matériaux et la Photonique) as previously described [22, 23]. Accelerated ^18^O ions (50 MeV/a, < 0.1 nA) were used at a dose rate of ~1 Gy/min which corresponded to a mean fluency of 1.22 × 10^5^ ions/ (cm^2^.s). A minimum of 30 s irradiation time was used in order to ensure a homogeneous dose on each sample. Except for survival curves, 2 Gy dose was used for all the samples.

Irradiation of 2D cells was performed as follows: cells were prepared similarly to X-ray irradiation protocol (see above). Subconfluent adherent cells were irradiated in an upright position at Room Temperature (Fig. 2b). Control flasks were mock-irradiated. Following irradiation, cells were maintained in the Tri-Gas Incubator (2 % O_2_) in fresh Growth Medium previously balanced with 2 % O_2_ for further cell survival experiments.

3DCM were maintained in a vertical position in a 2 mL polypropylene tube (Eppendorf®), with a sterile sample holder consisting of a glass cylinder as shown in Fig. 2b. The tube was filled with 3D Chondrocyte Medium previously balanced with 2 % O_2_ and irradiated in an upright position. In this configuration, collagen scaffolds are laid-out against the polypropylene tube thanks to the sample holder. Following irradiation, medium was changed and samples collected similarly to low-LET irradiated samples.

### LET distribution profile in the collagen scaffold

**Fig. 3:**
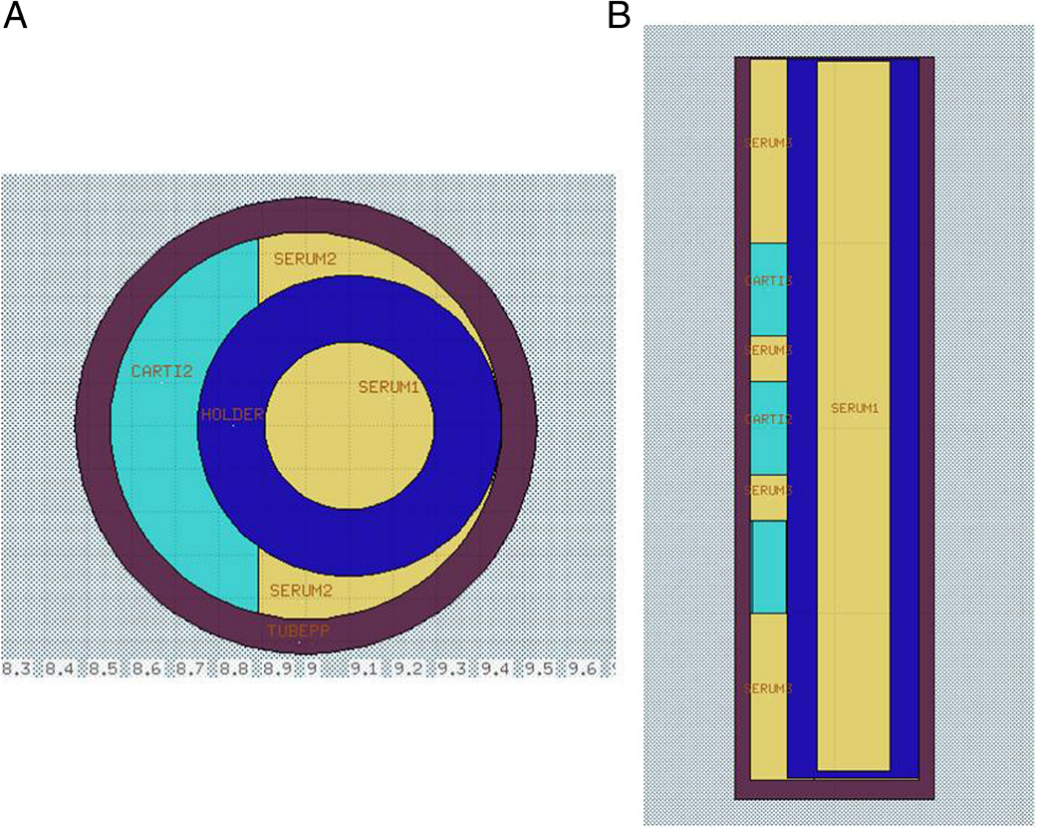
3DCM and polypropylene tube geometry used for FLUKA and PHITS calculations. Polypropylene tube (brown), curvature of the samples (light green) and holder positions (blue) were taken into account to reproduce the 3DCM configuration for the ion irradiation. Culture medium (mentioned as “serum”) is represented in yellow. Cross- (ZX, panel **a**) and longitudinal sections (XY, panel **b**) are represented

The assessment of the LET distribution profile for ^18^O ions is only available through a calculation code, based on the Monte-Carlo method, which simulates the transport of particles and their interactions into matter. Two calculation codes were used, with the appropriate description of heavy ion physics: FLUKA (FLUktuierende KAskade) [24, 25] from CERN (European Organization for Nuclear Research) and INFN (Istituto Nazionale di Fisica Nucleare) and PHITS (Particle and Heavy Ion Transport code System) [26] from JAERI (Japan Atomic Energy Research Institute). The irradiation beam line IRABAT was modelized, with its main features, such as splitter; iron window to produce X-rays for fluence estimation; mylar, gold and aluminium foils for ion distribution. A particular attention has been paid to reproduce the 3DCM configuration for the ion irradiation: polypropylene tube, curvature of the samples and holder position as shown in Fig. 3a and b. The same geometry was used for both Monte-Carlo codes. Energy cut-off, i.e. minimum energy for which a particle is tracked, was set to 1 keV. The LET distribution was calculated on the front side of the model (called proximal), on its rear side (called distal) and into the 3DCM model. In this study, only the LET distribution of incident ions was calculated. The LET from other particles, such as alpha particles, protons, delta electrons, fragmentation nuclei, were not calculated. Further calculations will be performed to determine the contribution of these particles [27] to the total dose into the 3DCM model.

### CFE: Colony Forming Efficiency (2D culture)

Clonogenic assessment in 2D was done by colony forming efficiency assay as previously described [28, 29]. Subconfluent 2D cells were irradiated as described above. The cells were left untreated from 12 to 16 h post-irradiation, then trypsinized, counted and plated in six-well plates (BD Falcon™) at two densities (100 or 1000 cells). The cells were grown for 12 days without medium change, then fixed and stained with crystal violet (3 % w/v in 20 % ethanol solution). Only colonies of 50 cells or more were scored. The experimental survival curve data were fitted to the linear quadratic equation [29]: − Ln (S) = αD + βD^2^. S is the surviving fraction at a given dose D. This calculation takes into account the mock-irradiated condition. α and β are constants determined by fitting the data to the model using the nonlinear regression program of the Prism software package (Graphpad Software, San Diego, CA). D_10_ (lethal dose for 10 % survival), D_37_ (lethal dose for 37 % survival) and SF_2_ (surviving fraction after a 2 Gy irradiation) values were determined from the fitted curve. Experiments were performed in triplicate and repeated from 2 to 6 times according to beam time availability. α and β are expressed as mean ± S.E.M.

Canonical Relative Biological Effectiveness (RBE) was calculated by dividing D_10_ of low-LET radiation by the corresponding parameter following high-LET irradiation. RBE was also calculated using D_37_.

### Cell toxicity assay

Cell toxicity was assessed on 3DCM culture medium using Toxilight™, a bioluminescent cytotoxicity assay designed to quantitatively measure the release of Adenylate Kinase (AK) from damaged cells. Manufacturer’s instructions were followed. Briefly, in a 96-white well plate (Greiner bio-one®), 20 μL of thawed cell culture supernatants were mixed to 100 μL/well of freshly prepared AK working solution. Plates were incubated for 5 min at room temperature before measurement. Flexstation 3 (Molecular Devices, Sunnyvale, USA) at the Proteogen plateform of Université Caen Basse-Normandie (UCBN) was used and programmed to 1 s integrated reading of appropriate wells. Digitonin detergent was used as a positive control (65 pM) as recommended by the manufacturer. Data were collected in RLU (Relative Light Unit).

### Immunohistochemistry-paraffin (IHC-p) staining

Formalin fixed 3DCM underwent a classical immunohistochemistry protocol (for paraffin sections), but manually performed to maintain scaffold integrity and avoid material loss. 3DCM were dehydrated in a graded series of ethanol on the first day and paraffin-soaked overnight. They were then paraffin-embedded in an upright position at the Pathology department of the CLCC François Baclesse. Eight μm sections were cut with a microtome and mounted on Superfrost™ Plus microscope slides, precisely, to avoid counting the same cells. The slides were dried overnight at 37 °C and stored at room temperature.

IHC staining was carried out overnight in a humidified chamber using monoclonal antibody directed against Ki67 diluted to 1/100 in 1 % BSA (Bovine Serum Albumin) in 0.5 % Tween PBS (TPBS). Then, 1 h incubation with HRP-conjugated anti-rabbit secondary antibody (diluted to 1/100 in 1 % BSA in TPBS) was done. Sections were then revealed with DAB and mounted using Eukitt mounting medium. No antigen retrieval was performed and as control, a slide without primary antibody incubation was realized.

The slides were observed with a Vanox-S Olympus light microscope (Tokyo, Japan) using a 40x lens. For each time point, around 200 nuclei were counted by the same experimenter, choosing 2 to 3 fields per slide (on a total of 5–10 slides). Only clearly stained cells were scored as DAB positives. Mock-irradiated sample is expressed as mean ± S.E.M from two independent experiments.

HES (Hematoxylin, Erythrosine, Safran) classical staining was also used to assess general organization of the collagen scaffold and cell distribution. Image acquisition was achieved, either with a Nikon Coolscope scanner (Tokyo, Japan) at the Pathology department of CLCC François Baclesse or Aperio Scancope CS scanner (Leica biosystems, Nussloch, Germany) at HIQ plateform of UCBN (Caen, France). Representative images were shown in the figures.

### Cell lysis protocol and western blotting

3DCM were disrupted using the following cell lysis protocol at 4 °C. Glass beads of 100 μm diameter (25 mg, Dominique Dutcher, Brumath, France) were added to a round-bottomed 2 mL Eppendorf tube and mixed with 40 μL of a freshly prepared lysis buffer [T-PER (1 mL), NaCl (850 mM), Halt Protease Inhibitors Cocktail (2X v/v), EDTA (2X v/v), Halt Phosphatase Inhibitors single-use Cocktail (2X v/v)]. One sample was then quickly thawed and mixed with lysis buffer and glass beads at 4 °C for 15 min using a disruptor GENIE™ (Dominique Dutcher). This cell lysis step was followed by addition of 10 μL Laemmli buffer (5X) and 5 min mix in the disruptor, to extract the proteins of the sample. This was followed by a protein denaturation step (sample heated twice at 100 °C for 5 min). After a quick centrifugation, 40 μL of the supernatant were collected. The sample underwent a second cycle of protein extraction-denaturation and 10 μL of extract were collected again. To estimate an extraction yield, the above protocol was performed twice on the same test sample, and the ratio of both extracts was calculated using ECL signal (the yield is expressed as mean ± S.E.M from two experiments).

Half of the extracted sample (25 μL) underwent SDS-Poly-Acrylamide 10 % Gel Electrophoresis (SDS-PAGE) and was transferred to a nitrocellulose membrane. Membranes were blocked for 1 h in 5 % milk in 0.05 % tween-TBS (TTBS) and incubated overnight at 4 °C with anti-H2AX phospho-serine 139 (gamma-H2AX) or anti-GAPDH as a loading control, both diluted at 1/1000 in 1 % milk TTBS. Membranes were then incubated for 1 h at room temperature with anti-mouse secondary peroxidase-conjugated antibodies (1/5000 diluted in 1 % milk TTBS). Detections were assessed on X-ray films (GE healthcare) using the ECL method. Image J software was used to quantify the non-saturated signals. Data were expressed as relative amount of gamma-H2AX compared to GAPDH. Evaluation of RBE (E_RBE_) was expressed as the relative amount of gamma-H2AX protein following high-LET irradiation divided by the same parameter post low-LET irradiation at the same time point.

Cell lysis and protein extraction using 2D cells were performed as described for 3DCM. Cell counting was used to adjust lysis and Laemmli buffer volumes. Around 0.25 million cells per sample were used to perform the western blot.

## Results and discussion

### SW1353 cells characterization

The therapeutic use of hadrons has mainly focused on low- and intermediate-grade CHS [2]. In this study, we focused on intermediate-grade (II) CHS as they show relative radioresistance to photons, a metastatic potential and high recurrence rate but still maintain a cartilage phenotype [30]. SW1353, JJ012 and CH3573 are currently the most characterized conventional grade-II CHS cell lines [31]. Among them, SW1353 is the most extensively used and is considered as the gold standard among other cells. Indeed, 142 articles were found in Pubmed library: [SW1353 OR HTB-94] compared to 39 articles for [JJ012] or 1 article for [CH3573]).

SW1353 cells were adapted from DMEM to a full standardized medium then amplified in a standard cell incubator (20 % O_2_ tension, normoxia). However, 3DCM were cultivated under 2 % oxygen tension in order to mimic human in situ microenvironment for cartilage [16, 17]. Such condition, referred here as physioxia, was then applied to 2D irradiations or experimental assays. Untreated SW1353 cells in 2D culture show a doubling time of 23 h and a usual cell cycle distribution with 42.2 % (±0.9), 35.2 % (±1.2) and 22.5 % (±0.8) of cells collected in G_0_/G_1_, S-phase and G2/M phase’s, respectively (Additional file 1: Figure S1).

3DCM were prepared by seeding 4 × 10^5^ cells in each scaffold and culturing them 7 days in physioxia as described before [15]. After 7 days of maturation, about 1.5 × 10^5^ of the seeded cells did not attach into the scaffold and were discarded by PBS washing. Cell distribution of attached cells throughout the scaffold was then analyzed. A representative image of a paraffin-embedded, transversally cut, HES colored slide of a 3DCM is shown in Fig. 1. The three magnified images (bottom) showed a homogenous cell distribution in the proximal (bottom, left), internal (bottom, middle) and distal (bottom, right) zones of the scaffold (Fig. 1). This was comparable with the cell distribution observed in 3DCaM seeded with AC using Scanning Electron Microscopy [15] or IHC and light microscopy [18]. Grade II CHS as SW1353 cell line show poor cellularity in histological sections [4]. Thus, under cell culture conditions described here, we were able to grow a homogeneous 3DCM miming an intermediate grade CHS tissue cellularity.

To evaluate the proliferation ratio of SW1353 cells in the 3DCM, we measured Ki67 index by IHC-p. Indeed, Ki67 index was previously described as a potential marker to assess tumor grade in CHS and determine the prognosis of patients with grade-II CHS [32]. This endpoint was also used to evaluate the impact of different drugs on CHS proliferation in a rat orthotopic CHS model [33]. Cell counting was done by a single experimenter and to assess intra-individual variability, the same slide was counted three times on different days with a resulting standard deviation of 2.4 % (Table 1). After seven days of culture into the collagen scaffold, the proliferation index of SW1353 cell line was 33 % ± 4 % (Table 2). This value is rather higher than the average index previously found in a retrospective study [32] on human CHS biopsy (14.7 % ± 4.4 % for grade-II tumors), although an extended custom range from 1.1 % to 50.2 % was described [32]. These discrepancies between human *in vivo* CHS and *in vitro* 3DCM may be explained by the cell line used but also by biopsy undefined genetic background and/or IHC technical issues. Furthermore, using primary human AC from two healthy male donors (38 and 51 years old), with non-apparent pathology, we prepared 3DCaM [15] with the same protocol described for the 3DCM. In comparison to 3DCM, a 2-fold inferior mean proliferation index was measured (17.5 % ± 4.5 %) in our conditions (Table 3). Such difference of proliferation indexes of primary chondrocytes and CHS cell line is consistent with human cartilage physiology [14, 16].

**Table 1:** Evaluation of the intra-individual counting variability of the Ki67 proliferation index in the 3DCM

Counting	Ki67 index (%)	Standard deviation (%)
Day 1	28.3	2.4
Day 2	33.1	
Day 3	30.8

**Table 2:** Ki67 proliferation index (%) 96 and 168 h following low-LET or high-LET irradiation

Radiation quality	Time post-irradiation (hrs)		
	0	96	168
Low-LET	33 ± 4	21	27
High-LET		45	43

**Table 3:** Ki67 proliferation index (%) in the 3DCaM using two healthy male donnors

	Ki67 proliferation index in the 3DCaM
Donnor 1 (38 years old)	13.0
Donnor 2 (51 years old)	22.0
Mean ± S.E.M	17.5 ± 4.5

### Irradiation set up, dosimetry and LET distribution

X-rays clonogenic assay experiments were performed with a 225 kV irradiator and a 15 MeV accelerator. Survival curve characteristics were almost identical with both devices (standard deviation < 10 %), a result which was previously described with different cell lines [34]. Subsequently, collected data were pooled. As X-rays beams were vertical, 2D culture flasks and 3DCM culture plates were maintained horizontal (Fig. 2a). On the contrary, heavy ion scanning beam from IRABAT line (GANIL) is horizontal (Fig. 2b). Thus, 2D culture flasks and tubes bearing 3DCM were then maintained in an upright position (Fig. 2b). However, in both conditions, flasks and 3DCM were fully filled with medium and irradiated in physioxia. Except for survival curves, SW1353 cells as 2D cultures or 3DCM were irradiated with 2 Gy of either X-rays (low-LET) or ^18^O ions (high-LET) radiation. These two conditions were chosen to mimic a canonical fraction of conventional radiotherapy (low-LET) versus a fraction of hadron-therapy with carbon ions (high-LET). Indeed, the LET of ^18^O accelerated ions (50 MeV/a) used in this study is approximatively of 103 ± 4 keV/μm which is comparable to the LET of SOBP of a carbon ions therapeutic beam [19].

**Fig. 4:**
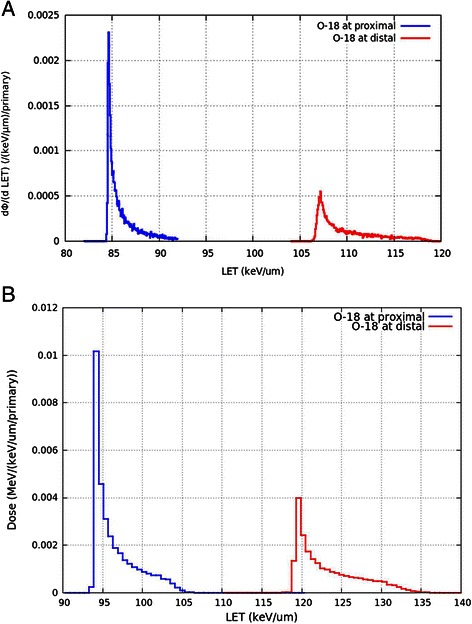
LET distribution profile of ^18^O ions in the proximal and distal zones of the 3DCM. FLUKA (panel **a**) and PHITS (panel **b**) calculation methods were used

Unlike X-rays, heavy-ions have a rapid energy deposition profile at the end of the track (Bragg peak). Calculations of the LET of ^18^O ions (50 MeV/a) for the proximal and distal zones and into the 3DCM were performed using FLUKA and PHITS calculation codes as described above. The corresponding values, shown in Table 4, are expressed as mean ± standard error. Using FLUKA, the calculated LET in the proximal and distal zones were 85.91 ± 0.38 and 109.82 ± 0.57 keV/μm, respectively. Using PHITS, the LET values of 96.27 ± 0.32 and 122.97 ± 0.67 keV/μm were calculated in the proximal and distal parts, respectively. These data reveal a difference of around 17 % between these two zones, regardless of the calculation method. Such variability is not surprising, considering the collagen scaffold thickness (2 mm). In addition, the proximal/distal LET distribution profile, shown in Fig. 4a and b, is also related to the curvature of the 3DCM set by the holder, which is used to maintain the scaffold in an upright position (Fig. 2b, Fig. 3a and b). This curvature has led to a variation in the thickness of 3DCM related to path of the incident ions. However, despite the scaffold thickness and irradiation geometry, only 17 % difference in LET was observed between front and rear sides of the 3DCM. These distribution profiles for the proximal and distal zones were not observed with a simulated flat geometry (not shown here). Moreover, as shown in Table 4, the mean LET into the 3DCM is 99.87 ± 0.21 keV/μm (range 85–120 keV/μm) using FLUKA, and 107.24 ± 0.11 keV/μm (range 95–135 keV/μm) using PHITS. Taken together, these simulation data show that cells are homogeneously irradiated by oxygen ions in this collagen scaffold, taking into account our irradiation geometry.

**Table 4:** LET values of ^18^O ions in the proximal and distal zones and into the 3DCM

Calculation code	LET (keV/μm)		
	Proximal zone	Into the 3DCM	Distal zone
FLUKA	85.91 ± 0.38	99.87 ± 0.21	109.82 ± 0.57
PHITS	96.27 ± 0.32	107.24 ± 0.11	122.97 ± 0.67

### Cell survival and proliferation post-irradiation

First, we estimated the clonogenic capacities (CFE) of SW1353 cells in standard 2D culture conditions (Table 5 and Additional file 2: Figure S2), as canonically performed in radiation-biology studies [35]. Exposure of 2D cells to low-LET and high-LET radiations revealed characteristic surviving fractions (Table 5). The low-LET survival parameters followed the two-hit target linear quadratic model (Additional file 2: Figure S2). In contrast, high-LET survival curves followed the one-hit target linear quadratic model (β = 0) (Additional file 2: Figure S2). As shown in Table 5, the value of α-component was higher with high-LET irradiation (2.567 ± 0.217 Gy^−1^) compared to low-LET irradiation (0.145 ± 0.005 Gy^−1^), both with a satisfying goodness of fit (R^2^ = 0.900 and R^2^ = 0.804, respectively). Extrapolated D_10_, D_37_ and SF_2_ were respectively 6.2 Gy, 3.6 Gy and 64.6 % for X-rays, and 0.9 Gy, 0.4 Gy and less than 1 % for ^18^O ions. Thus, not surprisingly, SW1353 in 2D culture were more resistant to low-LET radiation compared to high-LET. Canonical RBE calculation of ^18^O ions survival relative to X-rays (2D culture) shows a ratio of 6.8 (D_10_) whereas the ratio reach 9 when RBE is calculated from D_37_ (Table 5). These RBE values are concordant with a previous review of biological effectiveness of high-LET charged particles [19], considering a LET of 103 ± 4 keV/μm.

**Table 5:** Radiation survival curve characteristics for SW1353 cells cultured in 2D

Radiation quality	Energy	LET (keV/μm)	α (Gy^−1^)	β (Gy^−2^)	R^2^	D_10_ (Gy)	RBE_10_	D_37_ (Gy)	RBE_37_	SF_2_ (%)
X-rays	15 MeV 225 kV	~1	0.145 ± 0.036	0.037 ± 0.005	0.900	6.2	/	3.6	/	64.6
^18^O ion	50 MeV/a	103 ± 4	2.567 ± 0.217	0	0.804	0.9	6.8	0.4	9	<1

Clonogenic capacities were not feasible using the 3DCM, so to estimate cellular characteristics within this model, cell death and proliferation were measured using adapted experimental strategies. Indeed, combined treatment of the mature 3DCM with collagenase and Accutase™ or trypsine did not allow cell extraction in order to perform clonogenic assay. Chondrosarcoma cell lines are known for producing extracellular matrix [7] which may explain the difficulty in extracting them from the scaffold. Cell death of SW1353 in 3DCM following irradiation was then assessed with the Toxilight™ cytotoxicity assay as described above. The viable cell fraction was estimated using the ratio of luminescence produced by irradiated sample relative to mock-irradiated sample. We do not highlight any cellular toxicity induction 48 or 96 h following a 2 Gy irradiation in the 3DCM (ratio from 0.9 to 1.0), regardless of the radiation quality (low- or high-LET), while ratio from 65 pM digitonin treatment used as positive control scored 5.5 (Table 6).

**Table 6:** Cellular toxicity assay in 3DCM models following irradiation or digitonin treatment

	Mock (RLU)	Treated (RLU)	Ratio of luminescence relative to mock-treated sample
Digitonin 65 pM	4864	26490	5.5
Low-LET	7531	7193	1.0
High-LET	3313	2891	0.9

**Fig. 5:**
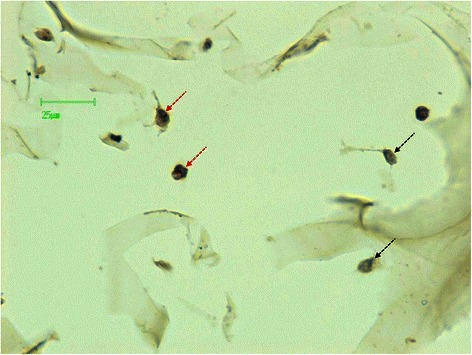
Ki67 immunostaining in the 3DCM. A representative cross-section of IHC-p stained 3DCM, using an antibody directed against Ki67 protein. Positive cells are indicated with red dotted arrows and negative cells with black dotted arrows. Counting positive cells served to assess proliferation index in the 3DCM

Proliferation index of SW1353 in 3DCM was measured by scoring Ki67 positive cells, as described above (Fig. 5, Table 2). The expression of human Ki67 protein is strictly associated with cell proliferation, present during all active phases of the cell cycle (G1, S, G2 and mitosis) but absent from quiescent cells (G_0_) [36]. However, it reflects the potential of cells to divide, but does not predict the actual division of these cells [37]. After 2 Gy of low- or high-LET radiation, this index was scored at day 4 and 7. We observed proliferation indexes of 21 % and 27 % in case of low-LET radiation, and 45 % and 43 % in case of high-LET, respectively (Table 1). This difference of proliferation index after low- or high-LET radiations may be explained by a higher number of cells arrested into the cell cycle (positive Ki67-cells arrested in G1, S, G2 or M phases) because of unrepaired clustered DNA damages. However, although these experiments will need to be deepened, we can hypothesize that there is still a fraction of cells in the irradiated 3DCM with a division potential. Indeed, it has been shown *in vivo* that such fraction of CHS cells in a quiescent step may contribute to the relative radioresistance of CHS to low-LET radiations [4].

### Post-irradiation gamma-H2AX repair kinetic

Reproducible and effective protein extraction from the 3DCM was a technical challenge, as cellular protein extraction from collagen scaffold was not efficient using a standard protocol. Thus, we developed a new protocol using a cell disruptor system and specific glass beads, as described above. This protocol allows us to calculate an extraction yield of 94 % (±4 %) from the quantification of western blot signal, showing the reproducibility and capacity of our protocol to extract and analyze low abundant proteins.

**Fig. 6:**
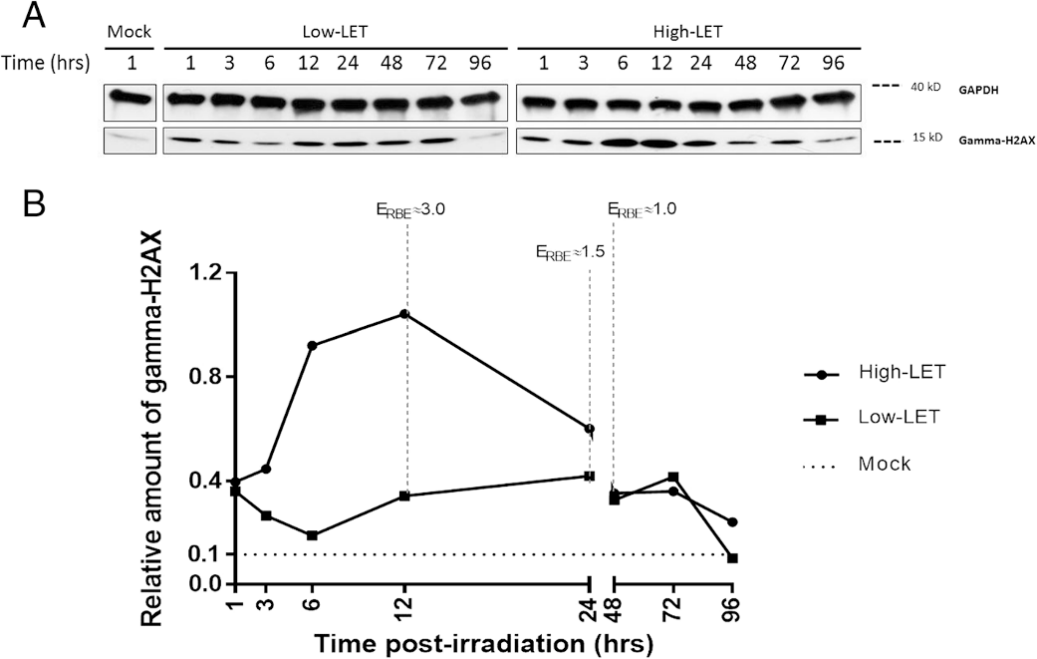
Post-irradiation gamma-H2AX repair kinetic in the 3DCM. Panel **a**: western blot analysis of gamma-H2AX, from 1 to 96 h following a 2 Gy low-LET or high-LET irradiation. GAPDH was used as a loading control. **b** Gamma-H2AX signal quantification normalized to GAPDH following a 2 Gy low-LET or high-LET irradiation. Image J software was used to analyze non-saturated signals. The non-cropped images are available in Additional file 3: Figure S3

The phosphorylated form of the histone H2AX (gamma-H2AX) is implicated in the DSB signaling and repair processes specially after IR exposure [38, 39]. H2AX phosphorylation was thereby chosen to estimate post-irradiation repair kinetic of SW1353 cells in the 3DCM. To do so, western blot was performed on 3DCM following 1 h to 96 h of a 2 Gy dose of low- or high-LET radiation (Fig. 6 and Additional file 3: Figure S3). The gamma-H2AX positive probing (15 kD), was measured with a modulated intensity throughout the kinetic. Using GAPDH as loading control (Fig. 6a), we measured a 4-fold gamma-H2AX induction with low-LET radiation, 1 h post-irradiation compared to mock-irradiated sample in 3DCM (Fig. 6b). However, following this induction, low-LET irradiated samples display a decrease in gamma-H2AX expression and regain mock-irradiated level 6 h post-exposure. Such data show that low-LET induced DNA strand breaks seem to be repaired quickly in 3DCM as previously described for 2D cell culture [38]. Proliferation-associated gamma-H2AX phosphorylation may explain the second wave of gamma-H2AX induction between 12 h and 72 h after low-LET exposure [40, 41].

On the contrary, following high-LET irradiation, gamma-H2AX induction still increases after 1 h post-irradiation to reach a maximal at 12 h with a 10-fold induction compared to the mock-irradiated sample (Fig. 6b). The gamma-H2AX expression then decreases slowly. This higher induction could be explained by the fact that high-LET radiations trigger more complex DNA damages that are harder to repair, compared to low-LET radiation as it has been already shown [42–44]. However, with 3DCM we show here that such higher gamma-H2AX induction is wider, meaning probably a delayed DNA repair kinetic due to our physioxic cell culture conditions. Furthermore, gamma-H2AX induction was also measured on 2D culture conditions at the same time points post-irradiation (Additional file 4: Figure S4 and Additional file 5: Figure S5). Using GAPDH as loading control, we measured ~ 3-fold gamma-H2AX induction with low-LET radiation, 1 h post-irradiation compared to mock-irradiated sample. Following this induction, low-LET irradiated samples display a two phase decrease with a fast first step until 3 h post-irradiation and then a slower decrease from 6 h to 24 h (Additional file 4: Figure S4). High-LET irradiated samples display a 3-fold gamma-H2AX induction 1 h post-irradiation, similarly to low-LET exposure. However, the decrease is very slow and only partial as a 2-fold induction is still measured 96 h after exposure (Additional file 5: Figure S5). Such delayed gamma-H2AX induction was recently described for high-LET radiation [45] and is consistent with well-known delayed DSB repair measured after high-LET exposure in 2D cell culture [46].

Finally, we propose a new evaluation marker of biological effectiveness named Evaluation of RBE (E_RBE_) by calculating the ratio of the gamma-H2AX induction following high-LET irradiation compared to low-LET as a function of time. Indeed, in the present study E_RBE_ at 12, 24 and 48 h following irradiation show a significant decrease as estimated respectively to 3.0, 1.5 and 1.0 in 3DCM (Fig. 6). This marker may be useful to monitor the impact of radiation quality in 3D models for whom collecting irradiated cells for canonical clonogenicity is impossible. Furthermore, analysis of E_RBE_ in a time course manner after radiation exposure may enlighten some undiscovered aspect of tissue response to hadron-therapy, especially for RT hypo-fractionation clinical protocols.

## Conclusions

In the present study we report, for the first time, a 3D physioxic CHS model applied to radiation-biology studies. We showed its usefulness for studying radiation quality impact on CHS in a controlled microenvironment that mimics *in vivo* tissue homeostasis after therapeutic radiation exposure. LET distribution calculation demonstrated a homogenously ions irradiation through the 3DCM. We describe here differential cellular responses (proliferation, gamma-H2AX induction kinetic) following low- versus high-LET radiation, using the same dose (2 Gy). The discrepancies found between 2D culture and 3DCM response to radiation, especially for high-LET exposure, may be explained by the reconstitution of a tissue-like microenvironment in 3DCM. Indeed, cells embedded in extracellular matrix change their whole metabolism [16] and may display specific delayed response as found for gamma-H2AX induction [41, 42]. Furthermore, high-LET irradiations of 3DCM show a particular gamma-H2AX induction, higher and longer than low-LET. Such specific pattern should be investigated in the future as 3D models may be used to assess new and more relevant indexes of radiation biological efficiency. Indeed, our 3DCM could be used as a validation tool for medical beams using carbon ions or protons in order to evaluate the RBE-LET dependence and measure the variation of RBE along the depth-dose profile of therapeutic proton or heavy ion beams. This issue was pointed out to be critical, at least in proton-therapy by Britten et al., 2013 [47].

Furthermore, the 3DCM strategy described here could also be applied to human AC or Mesenchymal Stem Cells (MSCs) for radiation quality impact investigations on normal cartilage or MSCs differentiation after therapeutic radiation exposure as it has been previously studied for low-LET radiations [48]. Such models could also be transplanted in nude mice, as it has been previously performed with AC [49].

Finally, in this study, we focused on targeted effects of IRs on CHS cultivated in 3D. However, non-targeted effects of IRs have been previously suggested to play a role in radiation-resistance of CHS to low-LET radiations [50]. Further studies with 3DCM using innovative tools will help understand the radiation-resistance of CHS in clinics [51].

## Additional files

Additional figure 1: **Cell cycle distribution of SW1353 cultured in 2D. Representative histogram, showing the cell cycle phases of SW1353 cultured in 2D.** G_0_/G_1_ phase is represented in blue, S phase in green and G_2_/M phase in pink. Percentage of cells in each phase is also reported. They represent the mean of four independent experiments ± SEM. (JPEG 12 kb)
Additional figure 2: **Radiation survival curve of SW1353 cultured in 2D.** SW1353 cells were irradiated with the indicated doses of X-rays (low-LET) or ^18^O ions (high-LET) then plated at low density, as described in methods. The quantification of the number of formed colonies after 11 days of incubation was used to calculate the surviving fraction relative to the mock-irradiation sample, at each radiation dose. The curve was fitted to the linear quadratic model. Symbols represent the mean of all replicates and error bars represent the SEMs. (JPEG 13 kb)
Additional figure 3: **Post-irradiation gamma-H2AX repair kinetic in the 3DCM (non-cropped images).** 3DCM were irradiated with a 2 Gy dose of low- or high-LET radiation as described above. 3DCM were collected 1 to 96 h following irradiation and stored at −80 °C. Mock-irradiated sample was treated the same way without being irradiated. The cell lysis protocol described above was used to prepare protein extracts. Half of each extract underwent SDS-PAGE (10 %) analysis. Low- (panels A, B) and high- (panels C, D) LET samples were loaded on two different gels, due to the limited number of lanes per gel. After the transfer step, membranes were cut just above 70 kD and incubated with the corresponding antibodies. Detections were assessed on X-ray films using two ECL reagents depending on signal intensity and the corresponding images are represented, with the ladder molecular weights (kD) on the right side of each image. The gamma-H2AX antibody was first used and positive probing observed at 15 kD; panel A (ECL classico, 30 s) and panel C (ECL classico, 30 s). The same membranes were, then, re-incubated with anti-GAPDH antibody and the positive probing observed between 35 kD and 40 kD: panel B (ECL classico, 30 s) and panel D (ECL crescendo, 5 min). The gamma-H2AX lane reappears at 15 kD due to the use of the same anti-mouse secondary antibody. (JPEG 194 kb)
Additional figure 4: **Post-irradiation gamma-H2AX repair kinetic in 2D, following low-LET irradiation.** Panel A: western blot analysis of gamma-H2AX, from 1 to 96 h following a 2 Gy low-LET irradiation. GAPDH was used as a loading control. (B) Gamma-H2AX signal quantification normalized to GAPDH following a 2 Gy low-LET irradiation. Image J software was used to analyze non-saturated signals. (JPEG 38 kb)
Additional figure 5: **Post-irradiation gamma-H2AX repair kinetic in 2D, following High-LET irradiation.** Panel A: western blot analysis of gamma-H2AX, from 1 to 96 h following a 2 Gy high-LET irradiation. GAPDH was used as a loading control. (B) Gamma-H2AX signal quantification normalized to GAPDH following a 2 Gy high-LET irradiation. Image J software was used to analyze non-saturated signals. (JPEG 34 kb)

## Abbreviations

2D: Two-dimensional
3D: Three-dimensional
3DCaM: 3D cartilage model
3DCM: 3D chondrosarcoma model
AC: Articular chondrocytes
AK: Adenylate kinase
BMP-2: Bone morphogenic protein-2
BSA: Bovine serum albumin
CFE: Colony forming efficiency
CHS: Chondrosarcoma
D_10_: Lethal dose for 10 % survival
D_37_: Lethal dose for 37 % survival
DAB: Diaminobenzidine
DMEM: Dulbecco's Modified Eagle's Medium
DSB: DNA Double strand break
ECL: Electrochemiluminescence
EDTA: Ethylene Diamine Tetraacetic Acid
E_RBE_: Evaluation of Relative Biological Effectiveness
FBS: Fetal Bovine Serum
GAPDH: Glyceraldehyde 3-Phosphate dehydrogenase
HES: Hematoxylin, erythrosine, safran
HRP: Horseradish Peroxidase
IHC-p: Immunohistochemistry-paraffin
IR: Ionizing radiation
LET: Linear energy transfer
MSCs: Mesenchymal stem cells
PBS: Phosphate-buffered saline
RBE: Relative biological effectiveness
RT: Radiation-therapy
SDS-PAGE: Sodium Dodecyl Sulfate-Poly-Acrylamide Gel Electrophoresis
S.E.M: Standard error on the mean
SF_2_: Surviving fraction after a 2 Gy irradiation
SOBP: Spread-out bragg peak
TBS: Tris-buffered saline
T-PER: Tissue protein extraction reagent
