# Supplementary figures and images for: In vitro engineering of human 3D chondrosarcoma: a preclinical model relevant for investigations of radiation quality impact
Source: BMC Cancer. 2015 Aug 8;15:579. doi: 10.1186/s12885-015-1590-5 (PMC4529727; doi:10.1186/s12885-015-1590-5)

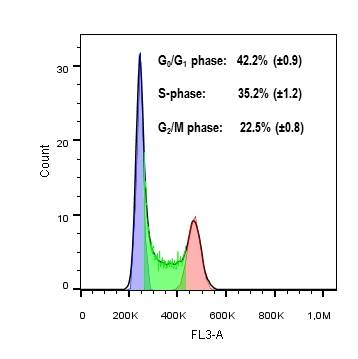

Supplement: Additional figure 1: — Cell cycle distribution of SW1353 cultured in 2D. Representative histogram, showing the cell cycle phases of SW1353 cultured in 2D. G0/G1 phase is represented in blue, S phase in green and G2/M phase in pink. Percentage of cells in each phase is also reported. They represent the mean of four independent experiments ± SEM. (JPEG 12 kb) [file 12885_2015_1590_MOESM1_ESM.jpeg]

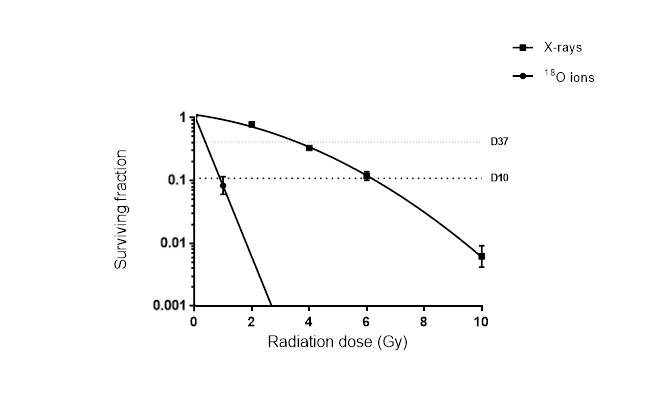

Supplement: Additional figure 2: — Radiation survival curve of SW1353 cultured in 2D. SW1353 cells were irradiated with the indicated doses of X-rays (low-LET) or 18O ions (high-LET) then plated at low density, as described in methods. The quantification of the number of formed colonies after 11 days of incubation was used to calculate the surviving fraction relative to the mock-irradiation sample, at each radiation dose. The curve was fitted to the linear quadratic model. Symbols represent the mean of all replicates and error bars represent the SEMs. (JPEG 13 kb) [file 12885_2015_1590_MOESM2_ESM.jpeg]

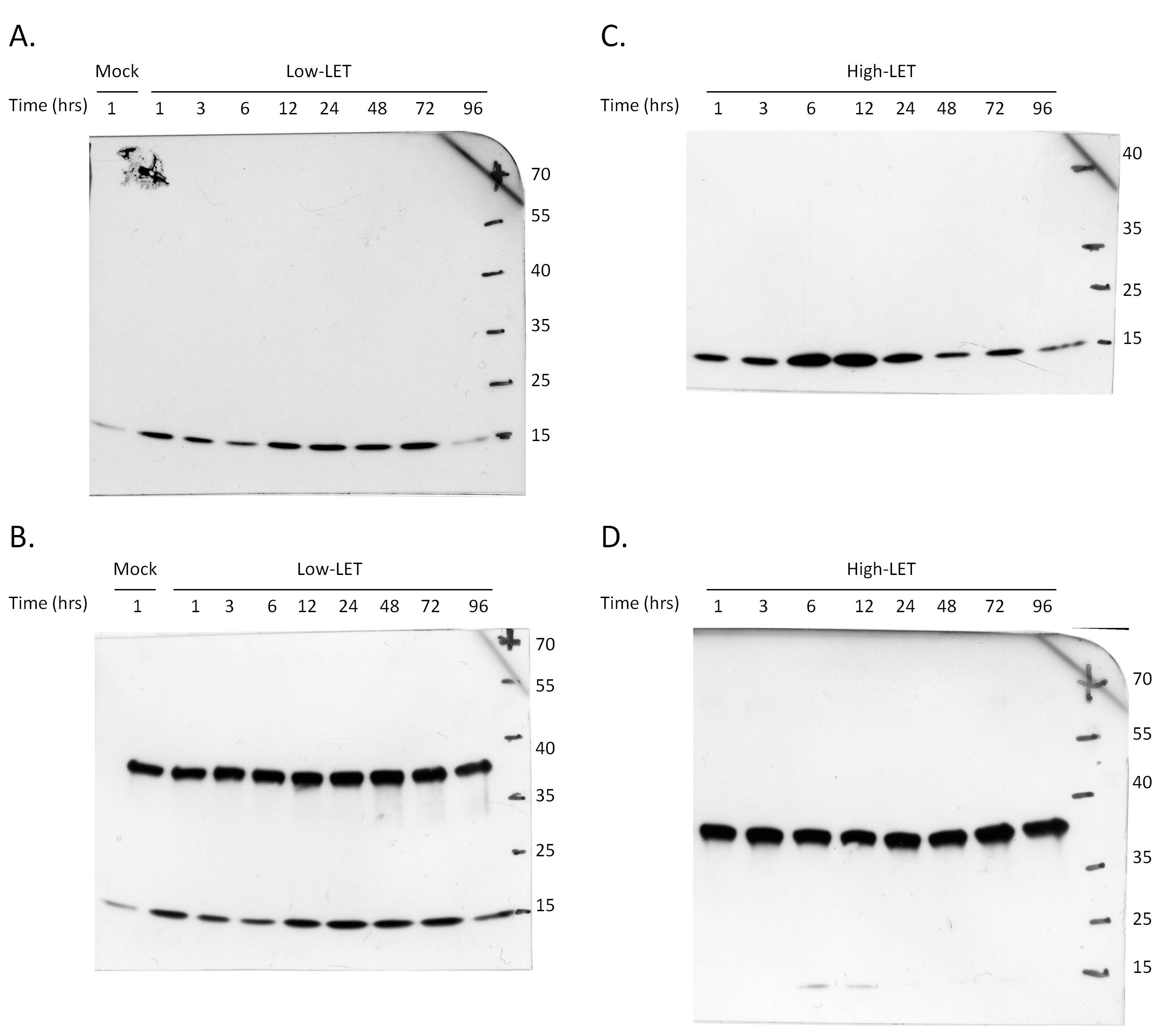

Supplement: Additional figure 3: — Post-irradiation gamma-H2AX repair kinetic in the 3DCM (non-cropped images). 3DCM were irradiated with a 2 Gy dose of low- or high-LET radiation as described above. 3DCM were collected 1 to 96 h following irradiation and stored at −80 °C. Mock-irradiated sample was treated the same way without being irradiated. The cell lysis protocol described above was used to prepare protein extracts. Half of each extract underwent SDS-PAGE (10 %) analysis. Low- (panels A, B) and high- (panels C, D) LET samples were loaded on two different gels, due to the limited number of lanes per gel. After the transfer step, membranes were cut just above 70 kD and incubated with the corresponding antibodies. Detections were assessed on X-ray films using two ECL reagents depending on signal intensity and the corresponding images are represented, with the ladder molecular weights (kD) on the right side of each image. The gamma-H2AX antibody was first used and positive probing observed at 15 kD; panel A (ECL classico, 30 s) and panel C (ECL classico, 30 s). The same membranes were, then, re-incubated with anti-GAPDH antibody and the positive probing observed between 35 kD and 40 kD: panel B (ECL classico, 30 s) and panel D (ECL crescendo, 5 min). The gamma-H2AX lane reappears at 15 kD due to the use of the same anti-mouse secondary antibody. (JPEG 194 kb) [file 12885_2015_1590_MOESM3_ESM.jpeg]

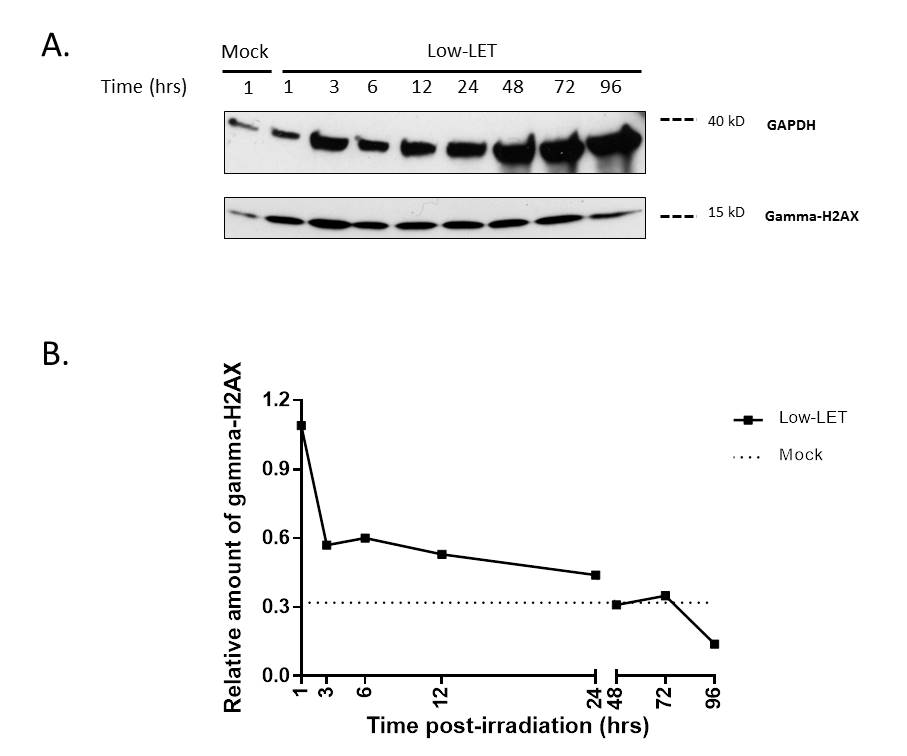

Supplement: Additional figure 4: — Post-irradiation gamma-H2AX repair kinetic in 2D, following low-LET irradiation. Panel A: western blot analysis of gamma-H2AX, from 1 to 96 h following a 2 Gy low-LET irradiation. GAPDH was used as a loading control. (B) Gamma-H2AX signal quantification normalized to GAPDH following a 2 Gy low-LET irradiation. Image J software was used to analyze non-saturated signals. (JPEG 38 kb) [file 12885_2015_1590_MOESM4_ESM.jpeg]

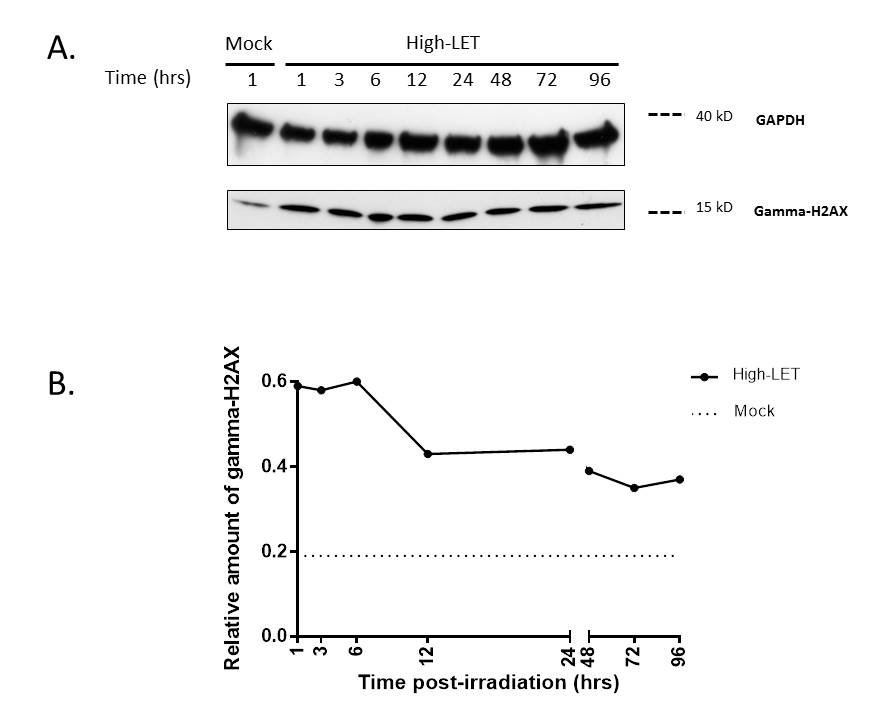

Supplement: Additional figure 5: — Post-irradiation gamma-H2AX repair kinetic in 2D, following High-LET irradiation. Panel A: western blot analysis of gamma-H2AX, from 1 to 96 h following a 2 Gy high-LET irradiation. GAPDH was used as a loading control. (B) Gamma-H2AX signal quantification normalized to GAPDH following a 2 Gy high-LET irradiation. Image J software was used to analyze non-saturated signals. (JPEG 34 kb) [file 12885_2015_1590_MOESM5_ESM.jpeg]
